# Establishment of an interdisciplinary curriculum in the core dental competence of “dental traumatology”

**DOI:** 10.3205/zma001837

**Published:** 2026-04-15

**Authors:** Derk Peters, Marius Crome, Kirstin Vach, Ingmar Staufenbiel, Alexander Rahman

**Affiliations:** 1 Hannover Medical School, Clinic for Conservative Dentistry, Periodontology and Preventive Dentistry, Hannover, Germany

**Keywords:** dental trauma, interdisciplinary teaching, learning gains, NKLZ, evaluation

## Abstract

**Objective:**

With the regulation for the reorganization of dental education, the legislature restructured dental studies and among other things, called for a strenghtening of interdisciplinary training. This requirement was the basis for the development of an interdisciplinary curriculum in which the topic of “dental traumatology” was to be taught across disciplines. It was investigated whether a measurable increase in learning could be achieved.

**Methodology:**

The newly designed curriculum was developed on the basis of the National Competence-Based Learning Objectives Catalogue for Dentistry (NKLZ) and was implemented in the summer semester of 2021. Due to the COVID-19 pandemic, the teaching module had to be modified significantly. In order to determine the students learning gains, an examination form was completed online before (T0) and after (T1) the curriculum. The exam paper consisted of knowledge questions and questions on two case vignettes.

**Results:**

The results showed a significant increase in learning in the knowledge questions (p<0.05). The case vignettes presented a heterogeneous picture. In case vignette V_(1)_, a significant increase in learning was achieved among students in the eighth (p=0.027) and tenth (p=0.022) semesters, but not among those in the sixth semester (p=0.323). In case vignette V_(2)_, a significant increase in learning was observed in the sixth (p=0.011) and tenth (p=0.003) semesters, but not in the eighth semester (p=0.298). The students evaluated the newly developed curriculum with a good rating.

**Conclusion:**

The interdisciplinary curriculum led to an increase in learning among the students. However, the heterogeneous results of the case vignettes show that even interdisciplinary lectures are not very efficient in acquiring the competence of “transfer thinking”. Nevertheless, interdisciplinary teaching formats should shape the future of dental education, but they should be supplemented by interactive elements.

## Zusammenfassung

**Zielsetzung:**

Der Gesetzgeber hat mit der Verordnung zur Neuregelung der zahnärztlichen Ausbildung das Studium der Zahnmedizin neustrukturiert und fordert u. a. eine Stärkung der fächerübergreifenden Ausbildung. Diese Forderung war Grundlage für die Entwicklung eines interdisziplinären Curriculums, bei dem das Thema „Dentale Traumatologie“ fächerübergreifend vermittelt werden sollte. Es wurde untersucht, ob ein messbarer Lernzuwachs erzielt werden konnte.

**Methodik:**

Das neu gestaltete Curriculum wurde auf Grundlage des Nationalen Kompetenzbasierten Lernzielkatalogs Zahnmedizin erstellt und fand im Sommersemester 2021 statt. Aufgrund der COVID-19-Pandemie musste das Lehrmodul stark modifiziert werden. Um den Lernzuwachs der Studierenden zu ermitteln, wurde ein Prüfungsbogen vor (T0) und nach (T1) der Veranstaltung online ausgefüllt. Der Prüfungsbogen umfasste reine Wissensfragen und (UV) Fragen zu zwei Fallvignetten.

**Ergebnisse:**

Die Ergebnisse zeigten einen signifikanten Lernzuwachs bei den Wissensfragen (p<0,05). Bei den Fallvignetten zeigte sich ein heterogenes Bild. Bei der Fallvignette V_1_ konnte bei Studierenden des achten (p=0,027) und des zehnten (p=0,022), nicht jedoch des sechsten Semesters (p=0,323) ein signifikanter Lernzuwachs erzielt werden. Bei der Fallvignette V_2_ zeigte sich ein signifikanter Lernzuwachs beim sechsten (p=0,011) und beim zehnten (p=0,003), nicht jedoch beim achten Semester (p=0,298). Die Studierenden evaluierten das neu entwickelte Curriculum mit der Note „gut“.

**Schlussfolgerung:**

Das interdisziplinäre Curriculum führte zu einem Lernzuwachs bei den Studierenden. Allerdings zeigen die heterogenen Ergebnisse der Fallvignetten, dass auch interdisziplinär gestaltete Frontalveranstaltungen wenig effizient bei dem Kompetenzerwerb „Transferdenken“ sind. Dennoch sollten interdisziplinäre Lehrformate die zahnmedizinische Lehre der Zukunft prägen, aber durch interaktive Elemente ergänzt werden.

## 1. Introduction

On 7 June 2019, the German Bundestag passed a new Dental Licensing Regulation (ZApprO), which calls for a complete restructuring and reorientation of the dentistry degree programme. Medical aspects, dental prevention and interdisciplinarity are to be strengthened [7]. Until now, dental education content has been taught predominantly in conventional curricula without an interdisciplinary concept, in accordance with the still valid licensing regulations (ZÄPrO of 1955). Taking dental traumatology as an example, surgical aspects were taught in the sixth semester, conservative and orthodontic aspects in the seventh, and prosthetic aspects in the eighth semester. This traditional separation of subjects often led to unnecessary repetition, but also to a lack of or difficulty in linking knowledge from different disciplines. In most faculties and universities, there was no coordination of content [35]. Until now, there has also been no catalogue of subjects in dentistry. The learning content was therefore often specified at the level of the chair [12]. A topic was discussed from the perspective of the individual subjects. Only by combining various theoretical and clinical content can a meaningful diagnosis and treatment plan be created, which ultimately also contributes to improving patient care. A study by Gräsel and Mandl showed that at the beginning of their clinical studies, the majority of students relied solely on data collection when making a diagnosis. The students were unable to form hypotheses based on the findings, relate them to each other and make possible diagnoses [14], [21], [31]. The aim of an interdisciplinary teaching concept should be to move students away from a passive consumer role and encourage them to actively participate in shaping their education. Obstacles to these concepts are often school-like and fixed teaching plans. The position paper of the German Society of Dentistry and Oral Medicine on the perspective of dentistry in 2030, on the other hand, is based on the principle of “EGNEZ: There is only ONE dentistry” and therefore supports the interdisciplinary reorientation of dentistry [13].

### 1.1. Traumatic dental injury

Traumatic dental injury is a common problem in dental emergency services. Almost every second child is affected during their lifetime, some even multiple times [5], [16], [19], [33]. While the tooth buds of the permanent dentition can be injured in the deciduous dentition, the effects on the permanent dentition are much more serious and have more serious consequences. Direct transmission of kinetic energy to the teeth can lead to fractures and dislocations, which can cause significant functional and/or aesthetic problems for those affected in the long term [1]. Although guidelines provide evidence-based recommendations for the immensely important measures of initial treatment, knowledge of these among the general dental profession is considered insufficient [22], [24], [34], [36]. The chance of success in preserving an avulsed tooth in the long term, for example, is determined by the extent of damage to the periodontal ligament (PDL) and bacterial contamination of the wound [3]. The measures taken by person at the scene of the accident therefore also have a significant impact on the long-term success of the treatment [23]. Through guideline-compliant, injury-specific initial care by the treating dentist, the damage caused can be kept as minimal as possible, and the traumatized teeth can be preserved in the long term in most cases [9]. If root growth is not complete, there is still a chance of pulp revascularisation in many dislocation injuries [2]. A good level of knowledge in this specialist area on the part of the dentist is therefore essential for the optimal treatment of dental trauma.

### 1.2. Needs analysis

As part of a needs analysis [20], the 2020 cohort was surveyed during their exam semester. The results of this survey showed a strong interest in linking interdisciplinary cases in clinical training. For this reason, the interdisciplinary curriculum “dental traumatology” presented here was developed as a pioneer for future interdisciplinary curricula. The aim of this measure was to lay a solid foundation of knowledge by involving all dental disciplines.

The original objective of the study was to investigate the influence of additional practice-oriented small group teaching on learning gains. Due to pandemic restrictions, small group teaching could not be carried out. For this reason, the research question had to be modified. The aim of the present study was to determine the learning gain, taking into account the subject semester in the newly developed interdisciplinary curriculum.

## 2. Methods

The study project was approved by the responsible ethics committee of the Medical University (No. 9297_BO_K_2020). The topic of “dental traumatology” was presented from the perspective of five different clinics (Clinic for Oral and Maxillofacial Surgery (MKG), Clinic for Conservative Dentistry, Periodontology and Preventive Dentistry (ZKO), Clinic for Dental Prosthetics (ZPW), Clinic for Orthodontics (ZOR) and Institute for Pharmacology). A matrix analysis was created to ensure that all teaching staff were involved in the objective decision-making process regarding teaching content and learning objectives [8].

Based on the matrix analysis, a blueprint and a Gantt chart were established for scheduling. In order to actively involve students in the development of learning objectives, students were also integrated into the working group. During the main phase, the learning objectives were formulated within the “dental traumatology” working group. Based on the learning objectives generated, presentations were created for the respective clinics, which were made available to students at the beginning of "trauma week" on the ILIAS learning platform (Integrated Learning, Information and Work Cooperation System: Peter L. Reichertz Institute, MHH). In the following team meetings, each lecturing member of the working group gave their presentation to avoid redundancy in content. Subsequently, the patient cases were selected and the learning content was discussed with the members of the working group.

In the resulting five-part lecture series, which were synoptically structured, each of the five clinics was allocated a time slot of 45 minutes, with the lectures focusing on selected patient cases. First, the Clinic for Conservative Dentistry, Periodontology and Preventive Dentistry addressed general aspects such as definition, aetiology, epidemiology, diagnostics and classification according to the type of injury, and then focused on conservative and endodontic measures for tooth preservation in therapy and aftercare. In contrast, the Clinic for Oral and Maxillofacial Surgery taught the diagnosis of cranio-cerebral trauma, immediate treatment of accompanying injuries, removal of teeth and fragments, splinting of loosened teeth using titanium trauma splints (TTS), and implantology concepts for rehabilitation after traumatic tooth loss. Immediate measures for the prosthetic restoration of fractured teeth or tooth loss, long-term prosthetic restoration after tooth loss, and aesthetic aspects in the anterior region were discussed in the context of prosthetics, while orthodontics focused on medium- and long-term orthodontic concepts for treatment after anterior tooth loss. Pharmacology lectured on local anaesthesia for immediate treatment of injuries, pain medication and the use of antibiotics, especially in the replantation of avulsed teeth. The practical relevance was established in the context of clinical cases, so that all possible measures for the selected patient cases, as well as their advantages and disadvantages, were discussed. The events took place simultaneously as part of the lecture “Dental, Oral and Maxillofacial Diseases Practicando and Auscultando” for students in their sixth, eighth and tenth semesters.

**Figure 1:**
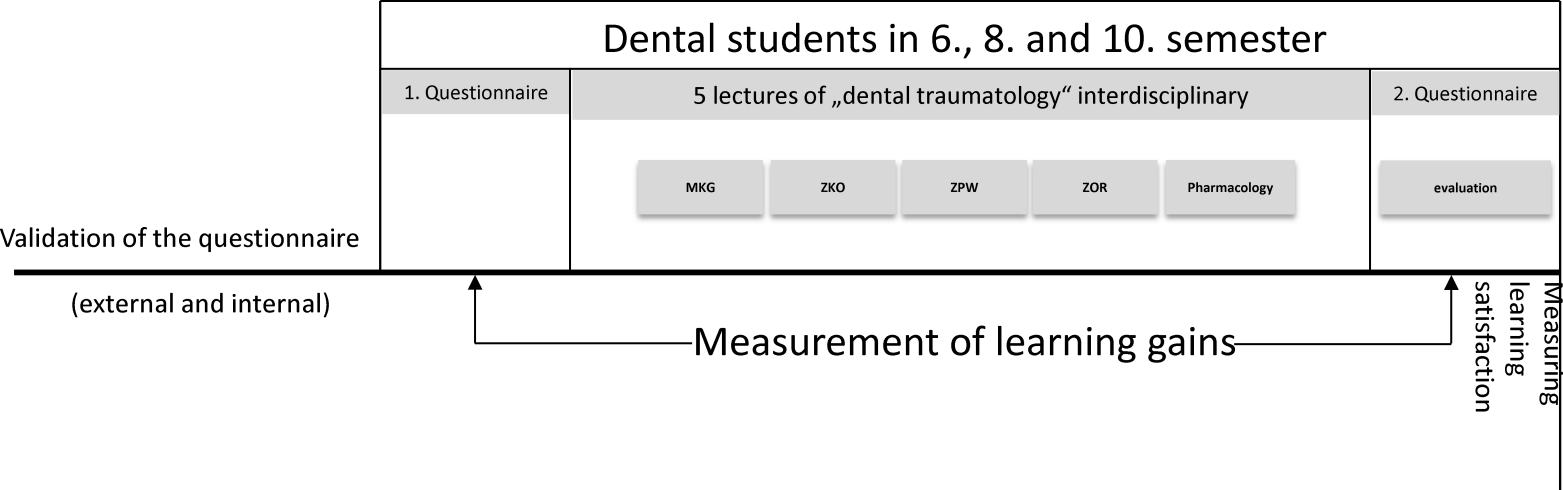
Chronological sequence of the newly developed teaching module

In advance, an examination paper was drafted with two case vignettes involving avulsions in the deciduous dentition (V_1_) and in the permanent dentition (V_2_), as well as 18 multiple-choice questions that tested factual knowledge on anterior tooth trauma according to the Miller pyramid [29]. The aspects of epidemiology, diagnostics, therapy and pharmacology relating to anterior tooth trauma were developed on the basis of the current literature and the National Competence-Based Learning Objectives Catalogue for Dentistry ([http://www.nklz.de/kataloge/nklz/lernziel/uebersicht], accessed on 10 March 2025). The examination paper was validated in advance by scientific staff at the Centre for Dental, Oral and Maxillofacial Medicine. All students in the sixth, eighth and tenth semesters were asked to complete the examination paper online before (T0) and after (T1) the course in order to measure their learning progress. To assess the level of knowledge, a total score (min: 0 – max: 152) was calculated as a global parameter based on the correct answers across all categories. In addition, questions were asked about two case vignettes. A maximum of 24 points could be achieved for case vignette 1 and a maximum of 30 points for case vignette 2. This resulted in a maximum total score of 206 points. The learning gain was calculated based on the difference in points between the surveys at time points T0 and T1. Another aspect that was asked about was the subjective self-assessment of the level of knowledge of dental traumatology before and after participating in the curriculum. At the second survey point (T1), an evaluation form was also completed (see figure 1 (Fig. 1)). Here, the curriculum was evaluated according to upper school points (0 to 15 points,0 points presented very poor and 15 points represented very good). In order to assign the information from the T0 and T1 questionnaires to the respective students, the questionnaires were coded using the first letter of the father's and mother's names, the first letter of the place of birth and the student's date of birth.

The original plan for the teaching module was to randomly assign students to a study group or control group at the beginning of the semester. Students in the control group were to be taught the interdisciplinary lecture content, while those in the study group were also to receive small-group instruction in seminar-sized classes with hands-on exercises. This practical instruction was to include a simulated anamnesis interview, a systematic assessment using the dental trauma assessment form of the German Society for Endodontology and Dental Traumatology (DGET), the practical performance of a pulpotomy on a fractured front tooth followed by adhesive restoration, and the repositioning of a dislocated tooth using a TTS. The learning success was to be additionally assessed in the study group with an OSCE (objective structured clinical examination) at the end of the semester [17].

Due to the contact and visitation bans in the sense of “social distancing” during the COVID-19 pandemic, it was not possible to conduct this small group teaching [32], so that the newly developed teaching module had to be held as an interdisciplinary, online-based frontal event.

### 2.1. Statistical analysis

The statistical analysis of the collected data was performed using the statistics software STATA (version 17.0; College Station, TX, USA). The mean values and standard deviations were determined for the descriptive analysis. The paired t-test was used to examine the learning success from T0 to T1 for each semester. A one-factor ANOVA was used to compare both the starting values and the changes between the three semesters. In subsequent pairwise comparisons, Scheffe's method was used to correct for multiple testing. The significance level was set at 5%. 

## 3. Results

A total of 231 students in the sixth, eighth and tenth semesters at Hannover Medical School were invited to participate in the teaching study. 149 exam papers were submitted. Of these, 53 questionnaires could not be evaluated due to missing information (e.g. no answers, only one exam paper submitted). The overall response rate was therefore 41.6% (96/231). Of these, 42 (out of 88) exam papers from the sixth semester, 29 (out of 70) from the eighth semester and 25 (out of 73) from the tenth semester could be included. The sample consisted of 65 (68%) female and 31 (32%) male students. The average age was 24.1±3.2 years.

In the first survey before the curriculum, differences between the semesters were observed. In terms of factual knowledge, sixth-semester students scored significantly lower (p<0.001) with 98.0±9.0 out of a possible 152 points than eighth-semester students (111.9±8.9 points) and tenth semester (118.0±9.7 points) students.

In case vignette V_1_, sixth-semester students achieved significantly fewer points (15.8±3.0 points; p=0.001) than eighth-semester students (18.5±2.6 points) and tenth semester (18.8±3 points) students prior to the curriculum (see table 1 (Tab. 1)). In case vignette V_2_, only one significant difference (p=0.018) could be demonstrated between the sixth (18.4±2.7 points) and tenth semesters (20.4±2.9 points).

**Figure 2:**
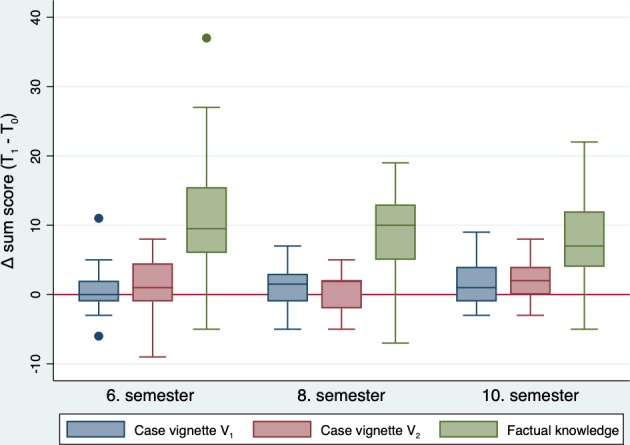
Difference in total score (T1-T0) for the two case vignettes and factual knowledge divided by semester presented as box plots.

In all three clinical semesters, a significant increase in learning was recorded in terms of factual knowledge (p=0.0001 in each case) (see figure 2 (Fig. 2)). There were no differences between the individual semesters (p=0.369) or between the genders.

In case vignette V_1_, a significant increase in learning was achieved among students in the eighth (p=0.027) and tenth semesters (p=0.022), but not among students in the sixth semester (p=0.323) (see table 1 (Tab. 1)). No significant difference was observed between the individual semesters (p=0.203). In case vignette V_2_ significant learning gains were observed in the sixth (p=0.011) and tenth semesters (p=0.003), but not in the eighth semester (p=0.298). Here, too, no significant difference between the semesters could be demonstrated (p=0.256).

In addition, students were asked to subjectively self-assess their level of knowledge before and after participation (see table 1 (Tab. 1)). Here, students in all semesters showed a significantly better self-assessment at T1 compared to T0 (p=0.001). In the students' evaluation based on upper secondary school points, the “dental traumatology” curriculum was rated 12.9±1.64 points.

## 4. Discussion

The potential loss of a front tooth not only means aesthetic and functional impairment for patients, who are usually still young, but can also have psychological consequences and entail financial costs [6]. Therefore, especially for these patients, the goal is to preserve a traumatised tooth (e.g. avulsed tooth) in the long term or until, for example, implant rehabilitation is possible. A good level of knowledge on the part of the dentist is essential for optimal initial care. Current literature has shown that there is a lack of basic knowledge about dental trauma among dentists [22], [34]. Therefore, the aim of this study was to determine the learning gains on the topic of “traumatic dental injury” among students in their clinical semesters before and after the implementation of a newly developed interdisciplinary curriculum. The statistical analysis showed that there was a significant increase in factual knowledge. It is well known that students are accustomed to learning facts due to their learning mentality. The challenge lies rather in applying factual knowledge to a clinical situation. Students must also be able to transfer their knowledge into clinical practice [11], [26], [28]. In addition, they should be able to explain and justify their clinical actions. This level of clinical competence (action competence) is also required of graduates in the National Competence-Based Learning Objectives Catalogue for Dentistry (NKLZ) ([http://www.nklz.de/kataloge/nklz/lernziel/uebersicht], accessed on 10 March 2025). Therefore, in the presentations of the individual clinics, emphasis was placed on presenting mainly clinical cases on the topic of “anterior tooth trauma”, as case-based learning is described in the literature as promising for clinical training [15]. However, this was only partially reflected in the results of the two clinical case vignettes. Here, the data was heterogeneous. In the first case vignette (V_1_), significant learning success was observed in the eighth and tenth semesters, but not in the sixth semester. One explanation for this could be differences in prior education. Unlike the students in the sixth semester, the students in the eighth and tenth semesters had already attended lectures on dental traumatology. In addition, the case vignettes were complex and designed with many questions, so that the students may have had difficulty maintaining their concentration over a longer period of time [18], [30]. Furthermore, it is conceivable that due to the lack of grading, the students’ motivation to work on the complex case vignettes with the appropriate care was low [27]. The sometimes large standard deviation in the students’ learning success could be an indication that the students’ knowledge and learning methods differed greatly in some cases. Another approach discussed in the literature on transfer thinking is a lack of problem-solving skills that cannot be compensated for with existing factual knowledge [10]. It is imperative to provide students with sufficient practical skills in addition to factual knowledge. This point is crucial for the classification of a clinical case and the subsequent therapy decision [4]. Unfortunately, shortly before the start of the study, an extension of the contact and visitation bans (COVID-19 pandemic) was ordered [32]. The students were to be divided into a study group and a control group each semester. In the study groups, diagnoses and treatment plans were to be developed on the basis of clinical cases and supervised by a research assistant. In addition, practical skills were to be taught. At the end of the teaching unit, an OSCE examination was planned for the study group. However, due to the extended lockdown, it was not possible to carry out the originally planned study. As a result, only frontal lectures could be held online. Even though these were interdisciplinary in nature, they did not improve transfer thinking or practical skills. Interaction is needed here. The students were asked to assess their own subjective level of knowledge before and after the curriculum. They tended to rate their knowledge significantly higher after the curriculum. However, this self-assessment must be viewed critically and could lead to students overestimating their abilities. This effect is known as the Dunning-Kruger effect [25]. In order for them to be able to assess their clinical skills, these would also have had to be taught and tested.

This study showed that learning gains were achieved in all semesters with regard to questions on factual knowledge. However, abstracting this knowledge and transferring it to a clinical situation still seems to pose a challenge for students. When assessing learning gains, the quality of learning and the form of competence acquisition play a major role.

### 4.1. Limitations of the study 

Firstly, it should be noted that with 96 participating students (out of a total of 231 students), less than half of the students took part in the survey. The small number of cases does not allow for any fundamental new findings in teaching research. Since the survey was deliberately conducted on a selected sample of dental students at Hannover Medical School, it is not possible to generalize or transfer the results to other locations. It should also be noted that the questionnaires were completed at different locations using mobile devices at both points in time. The environment was therefore not controlled, distractions etc. may have occurred, and the students were unable to ask any questions. In addition, no dropout analysis was carried out, i.e. the characteristics of the participants who did not take part in the survey were not examined.

## 5. Conclusions

The newly developed interdisciplinary curriculum “dental traumatology” proved to be successful in terms of learning outcomes regarding factual knowledge and learning satisfaction. However, the results of the study also show that frontal teaching cannot strengthen students' transfer thinking or practical skills. A successful learning outcome in these competencies can only be achieved through interactive small group teaching and practical exercises.

## Notes

### Funding

The study was funded by the Lower Saxony Ministry of Science and Culture as a part of its Innovative Teaching and Learning Concepts (Innovation Plus) programme.

### Authors

The authors Ingmar Staufenbiel and Alexander Rahman are equal senior authors.

### Authors’ ORCIDs

- Derk Peters: [0009-0000-9124-8666]
- Kirstin Vach: [0000-0001-9278-2203]
- Ingmar Staufenbiel: [0000-0002-7155-8402]
- Alexander Rahman: [0000-0001-9491-3107]

## Competing interests

The authors declare that they have no competing interests. 

## Figures and Tables

**Table 1:**
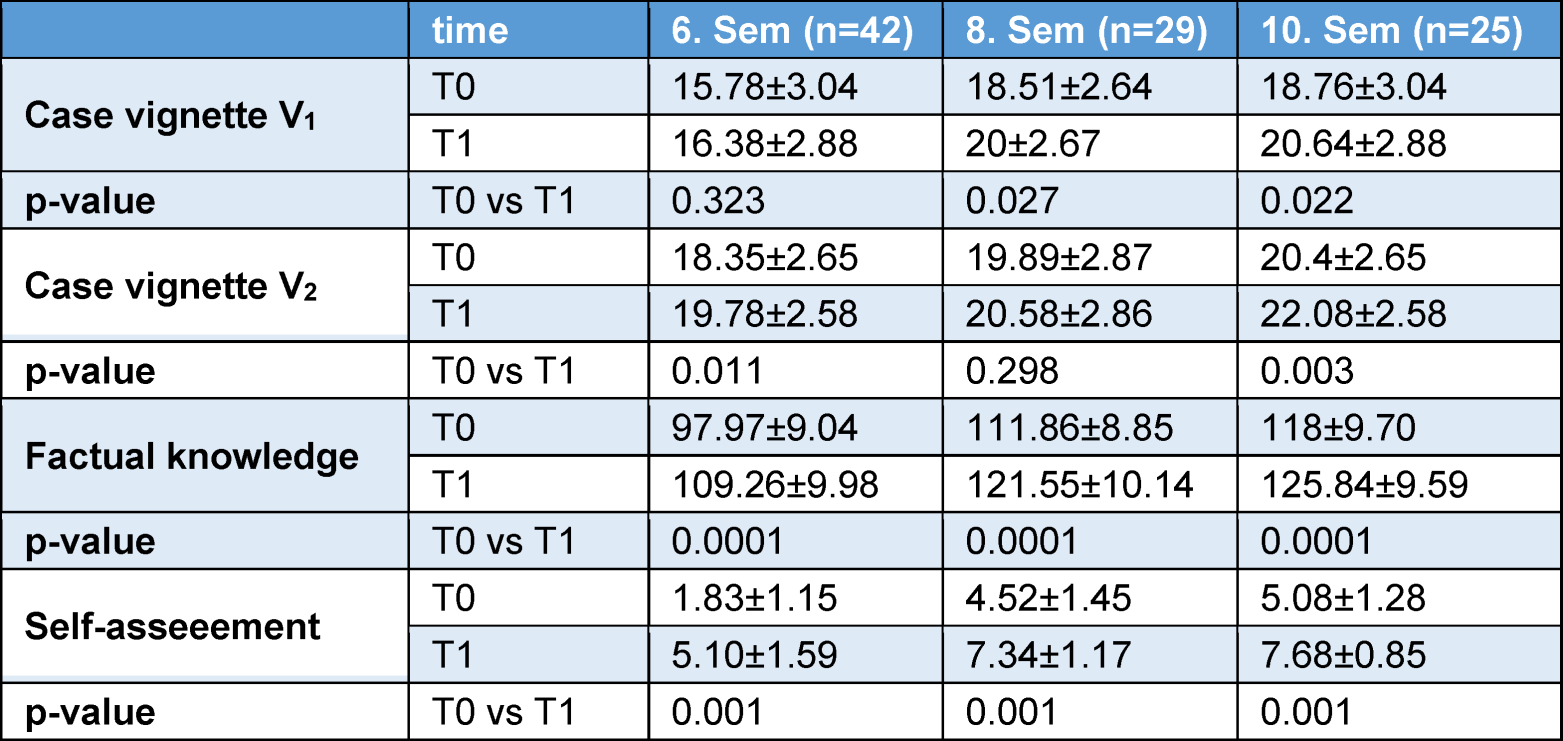
Mean values±standard deviations for case vignettes V_1_, V_2_ and factual knowledge for the time points before intervention (T0) and after intervention (T1), comparison of mean values between T0 and T1 within a semester (paired T-test).
